# Feasibility study of using simultaneous multi-slice RESOLVE diffusion weighted imaging to assess parotid gland tumors: comparison with conventional RESOLVE diffusion weighted imaging

**DOI:** 10.1186/s12880-020-00492-1

**Published:** 2020-08-06

**Authors:** Jia-Suo Jiang, Liu-Ning Zhu, Qian Wu, Yi Sun, Wei Liu, Xiao-Quan Xu, Fei-Yun Wu

**Affiliations:** 1 Department of Radiology, The First Affiliated Hospital of Nanjing Medical University, No. 300, Guangzhou Rd, Nanjing, China; 2 Department of Stomatology, The First Affiliated Hospital of Nanjing Medical University, Nanjing, China; 3 MR Collaboration, Siemens Healthcare Ltd., Shanghai, China; 4 Siemens Shenzhen Magnetic Resonance Ltd., Shenzhen, China

**Keywords:** Parotid gland, Magnetic resonance imaging, Diffusion-weighted imaging, Readout segmentation of long variable echo-trains, Simultaneous multi-slice

## Abstract

**Background:**

To evaluate the feasibility of using simultaneous multi-slice (SMS) readout segmentation of long variable echo-trains (RESOLVE) diffusion-weighted imaging (DWI) to assess parotid gland tumors, compared with conventional RESOLVE DWI.

**Methods:**

From September 2018 to December 2018, 20 consecutive patients with parotid tumors who underwent MRI scan for pre-surgery evaluation were enrolled. SMS-RESOLVE DWI and conventional RESOLVE DWI were scanned with matched imaging parameters, respectively. The scan time of two DWI sequences was recorded. Qualitative (anatomical structure differentiation, lesion display, artifact, and overall image quality) and quantitative (apparent diffusion coefficient, ADC; ratio of signal-to-noise ratio, SNR ratio; ratio of contrast-to-noise ratio, CNR ratio) assessments of image quality were performed, and compared between SMS-RESOLVE DWI and conventional RESOLVE DWI by using Paired *t*-test. Two-sided *P* value less than 0.05 indicated significant difference.

**Results:**

The scan time was 3 min and 41 s for SMS-RESOLVE DWI, and 5 min and 46 s for conventional RESOLVE DWI. SMS-RESOLVE DWI produced similar qualitative image quality with RESOLVE DWI (anatomical structure differentiation, *P* = 0.164; lesion display, *P* = 0.193; artifact, *P* = 0.330; overall image quality, *P* = 0.083). Meanwhile, there were no significant difference on ADC_Lesion_ (*P* = 0.298), ADC_Masseter_ (*P* = 0.122), SNR ratio (*P* = 0.584) and CNR ratio (*P* = 0.217) between two DWI sequences.

**Conclusion:**

Compared with conventional RESOLVE DWI, SMS-RESOLVE DWI could provide comparable image quality using markedly reduced scan time. SMS could increase the clinical usability of RESOLVE technique for DWI of parotid gland.

## Background

Diffusion weighted imaging (DWI) is a widely-used technique for evaluating the rate of microscopic water diffusion in tissues. Its derived apparent diffusion coefficient (ADC) has been accepted as an important diagnostic marker for characterizing parotid gland tumors [1–6]. In clinical practice, single-shot echo-planar imaging (SS-EPI) is commonly used for DWI scan. Despite the scan speed is fast, SS-EPI based DWI is prone to geometric distortions, image blurring and susceptibility artifacts [7]. Recently, readout segmentation of long variable echo-trains (RESOLVE) based DWI attracts increasing attention. It can significantly reduce distortions and artifacts, however it was restricted for the long scanning time [7–12].

Recently, simultaneous multi-slice (SMS) imaging with blipped ‘Controlled aliasing in parallel imaging results in higher acceleration’ (blipped-CAIPIRINHA) technique attracts increasing attention [13–21]. In this approach, multiple slices are excited by a single radio frequency pulse and spatially shifted in the phase encoding direction to improve the utilization of coil sensitivity information. In the end, the slice-GRAPPA (Generalized Auto Calibrating Partially Parallel Acquisitions) is used to separate the collapsed slices [13]. Till now, few studies involving breast and kidney tried to combine SMS and RESOLVE DWI for reducing scanning time [14, 19], while study evaluating the feasibility of SMS-RESOLVE DWI in assessing the parotid gland tumors is still lacked.

Therefore, the purpose of present study is to evaluate the feasibility of SMS-RESOLVE DWI in assessing parotid gland tumors, compared with conventional RESOLVE DWI technique.

## Methods

### Patients

This prospective study was approved by the Ethics Committee of our hospital, and written informed consents were obtained from all patients. From September 2018 to November 2018, twenty consecutive patients (11 males and 9 females; mean age, 55.0 ± 14.0 years; range, 29–76 years) with parotid tumors underwent MRI examination for pre-surgery evaluation. The twenty patients include 8 patients with pleomorphic adenomas, 7 patients with warthin tumors, 3 patients with lymphomas, one patient with mucoepidermoid carcinoma and one patient with lymphoepithelioma.

### Image acquisition

A 3.0-T MR scanner (MAGNETOM Skyra, Siemens Healthcare, Erlangen, Germany) equipped with a twenty-channel head/neck coil was used in this study. Patients rested in the supine position. Imaging protocols contained an unenhanced axial T1-weighted imaging (repetition time [TR]/echo time [TE], 801/6.7 ms) and axial T2-weighted imaging (TR/TE, 4000/85 ms) with fat saturation. Two sets of DWI sequences (a prototype sequence SMS-RESOLVE and a product sequence RESOLVE) were performed with comparable imaging parameters. Detailed imaging parameters of these two sequences were summarized in Table 1.

**Table 1:** Imaging parameters of SMS-RESOLVE DWI and RESOLVE DWI

Parameter	SMS-RESOLVE	RESOLVE
TR (ms)	4000	6750
TE (ms)	72	69
Slices	30	30
Slice thickness (mm)	3	3
Distance factor (%)	10	10
Voxel size (mm^3^)	1.1 × 1.1 × 3.0	1.1 × 1.1 × 3.0
Fat suppression	Fat sat	Fat sat
Readout segments	5	5
Readout partial Fourier acquisition	7/8	7/8
FOV (mm^2^)	220 × 220	220 × 220
Averages	b_0_ = 2, b_1000_ = 2	b_0_ = 2, b_1000_ = 2
GRAPPA acceleration factor	2	2
Slice acceleration factor	2	–
Acquisition time (min:s)	3:41	5:46

*SMS* simultaneous multi-slice, *RESOLVE* readout segmentation of long variable echo-trains, *DWI* diffusion-weighted imaging, *TR* repetition time, *TE* echo time, *FOV* field of view, *GRAPPA* generalized auto calibrating partially parallel acquisitions.

### Qualitative comparisons of image quality

Qualitative assessment of RESOLVE DWI and SMS-RESOLVE DWI sequences was performed based on the following four aspects and a 4-point criteria: anatomical structure differentiation (1: poor, 2: acceptable, 3:good, 4: excellent), lesion display (1: vaguely seen, 2: identifiable, 3: blurry borders, 4: sharp borders), artifact (1: definitely confounding interpretation, 2: present, but little impact on interpretation, 3:faint, 4: no artifact), and overall image quality (1: poor, 2: acceptable, 3: good, 4: excellent).

### Quantitative comparisons of image quality

**Fig. 1:**
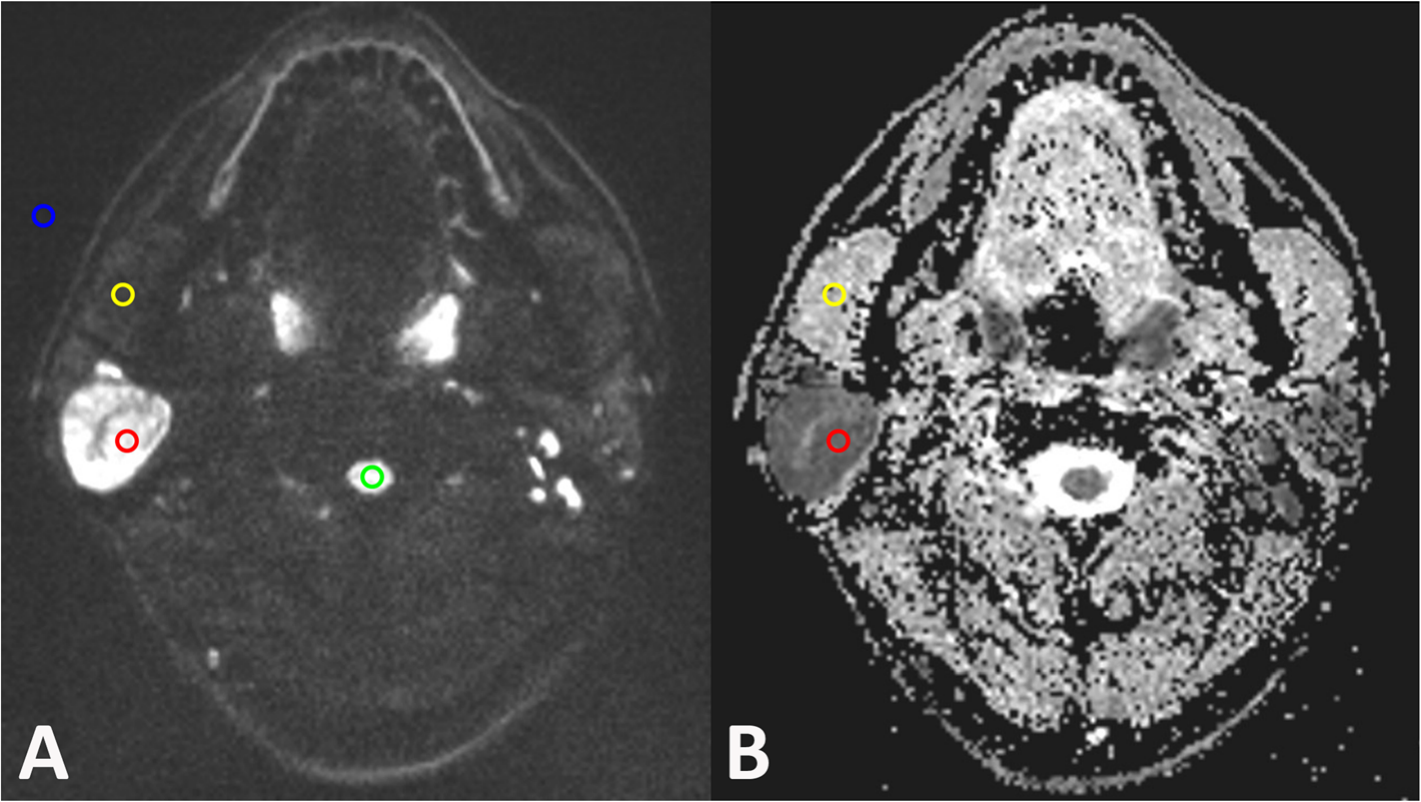
Schematic diagram of the placements of ROIs. Based on the DW (b_1000_) image (**a**) of a 47-year-old patient with a warthin tumor in the right parotid gland, round regions of interest (ROIs) were drawn in parotid tumors (red), ipsilateral masseter muscle (yellow), ipsilateral spinal cord (green) and background (blue), respectively. The central cystic area was avoided for measurement. Then, the same ROIs were copied on the ADC map (**b**), and the mean ADC value of tumor and masseter were measured

Quantitative assessments were performed on the dedicated post-processing workstation (MAGNETOM Skyra, Siemens Healthcare, Erlangen, Germany). The slice in which the parotid gland tumor showed the largest area was chosen for quantitative analysis. For multicenter lesions, the larger one was selected for assessment. Based on the DW (b_1000_) image, round regions of interest (ROIs) with area about 15 mm^2^ (14.90 ± 0.61 mm^2^) were drawn in parotid tumors, ipsilateral masseter muscle, ipsilateral spinal cord and background, respectively. ROIs within the tumors were placed by avoiding possible cystic, necrotic and hemorrhagic portion. Schematic diagram of the ROIs placements was displayed in Fig. 1. After the ROIs were placed, the mean signal intensity of the lesion (S_Lesion_), signal intensity of the masseters (S_Masseter_), signal intensity of the spinal cord (S_Spinal_), standard deviation of the background (σ_Background_), standard deviation of the masseter (σ_Masseter_), standard deviation of the spinal cord (σ_Spinal_) and standard deviation of the lesion (σ_Lesion_) were automatically acquired. Then, the signal-to-noise (SNR) was calculated as the ratio between S_Lesion_ or S_Spinal_ and σ_Background_ (SNR lesion = S_Lesion_/σ_Background_; SNR spinal cord = S_Spinal_/σ_Background_) [22]. SNR ratio was calculated using the following formula: SNR ratio = SNR lesion / SNR spinal cord. Contrast-to-noise (CNR) was calculated using the following formula: $CNR\kern0.5em \mathrm{lesion}\kern0.5em =\frac{S_{L\mathrm{esion}}-{S}_{\mathrm{Masseter}}}{\sqrt{{\upsigma_{\mathrm{Lesion}}}^2+{\upsigma_{\mathrm{Masseter}}}^2}}$; $CNR\kern0.5em \mathrm{spinal}\kern0.5em cord\kern0.5em =\frac{S_{S\mathrm{pinal}}-{S}_{Masseter}}{\sqrt{{\sigma_{Spinal}}^2+{\sigma_{Masseter}}^2}}$ [22]. CNR ratio was calculated using the following formula: CNR ratio = CNR lesion / CNR spinal cord. After the same ROIs were copied on the ADC map, the mean ADC value of tumor and masseter were automatically achieved, and denoted as ADC_Lesion_ and ADC_Masseter_, respectively.

Two radiologists (reader 1: with 2 years of experience; reader 2: with 6 years of experience) performed the qualitative and quantitative analysis independently. The mean values of two readers were applied to further statistical analyses.

### Statistical analysis

Continuous parameters were expressed as mean ± standard deviation. Kolmogorov-Smirnov test was used for normally distributed analysis. Paired *t*-test was used to compare the difference of qualitative scores, ADC_Lesion_, ADC_Masseter_, SNR ratio and CNR ratio between SMS-RESOLVE DWI and RESOLVE DWI. Intra-class correlation coefficient (ICC) with 95% confidence interval (CI) was used to assess the inter-reader agreement of ADC, SNR ratio and CNR ratio measurements. The inter-reader agreement of the qualitative scores was assessed with kappa analysis. ICC and kappa values were interpreted as follows: (≤ 0.40, poor; 0.41–0.60, moderate; 0.61–0.80, good; ≥ 0.81, excellent) [11]. All statistical analyses were performed by using two commercially available software packages (SPSS version 22.0, IBM Corp., Armonk, NY; MedCalc 15.0, Mariakerke, Belgium). Two-sided *P* value less than 0.05 indicated significant difference.

## Results

### Scan time

The scan time for SMS-RESOLVE DWI was 3 min and 41 s. The scan time for RESOLVE DWI was 5 min and 46 s.

### Qualitative comparisons

The difference was not statistically significant for all four qualitative score items between SMS-RESOLVE DWI and conventional RESOLVE DWI (anatomical structure differentiation, 3.05 ± 0.69 vs 3.15 ± 0.67, *P* = 0.164; lesion display, 3.55 ± 0.69 vs 3.70 ± 0.66, *P* = 0.193; artifact, 2.95 ± 0.51 vs 2.90 ± 0.55, *P* = 0.330; overall image quality, 3.05 ± 0.76 vs 3.20 ± 0.77, *P* = 0.083) (Table 2).

**Table 2:** Qualitative comparisons of image quality between SMS-RESOLVE DWI and RESOLVE DWI

Qualitative parameters	SMS-RESOLVE	RESOLVE	P	kappa	
				SMS-RESOLVE	RESOLVE
anatomical structure differentiation	3.05 ± 0.69	3.15 ± 0.67	0.164	0.74	0.83
lesion display	3.55 ± 0.69	3.70 ± 0.66	0.193	0.79	0.71
artifact	2.95 ± 0.51	2.90 ± 0.55	0.330	0.79	0.78
overall image quality	3.05 ± 0.76	3.20 ± 0.77	0.083	0.84	0.92

*SMS* simultaneous multi-slice, *RESOLVE* readout segmentation of long variable echo-trains, *DWI* diffusion-weighted imaging.

Data are expressed as mean ± standard deviation.

The Kappa values of the four qualitative assessments bases on SMS-RESOLVE DWI ranged from 0.74–0.84, while those based on conventional RESOLVE DWI ranged from 0.71–0.92 (Table 2).

### Quantitative comparisons

There were no significant differences on ADC_Lesion_ (0.91 ± 0.37 vs 0.92 ± 0.37, *P* = 0.298), ADC_Masseter_ (1.41 ± 0.17 vs 1.55 ± 0.11, *P* = 0.122), SNR ratio (1.92 ± 0.36 vs 1.77 ± 0.74, *P* = 0.584) and CNR ratio (1.28 ± 0.59 vs 1.11 ± 0.58, *P* = 0.217) between SMS-RESOLVE DWI and conventional RESOLVE DWI (Table 3).

**Table 3:** Quantitative comparisons of image quality between SMS-RESOLVE DWI and RESOLVE DWI

Quantitative parameters	SMS-RESOLVE	RESOLVE	P	ICC	
				SMS-RESOLVE	RESOLVE
ADC_Lesion_	0.91 ± 0.37	0.92 ± 0.37	0.298	0.89	0.86
ADC_Masseter_	1.41 ± 0.17	1.55 ± 0.11	0.122	0.87	0.74
SNR ratio	1.92 ± 0.36	1.77 ± 0.74	0.584	0.75	0.78
CNR ratio	1.28 ± 0.59	1.11 ± 0.58	0.217	0.83	0.88

*SMS* simultaneous multi-slice, *RESOLVE* readout segmentation of long variable echo-trains, *DWI* diffusion-weighted imaging, *ADC* apparent diffusion coefficient, *SNR* signal-to-noise ratio, *CNR* contrast-to-noise ratio, *ICC* intra-class correlation coefficient.

Unit of ADC is ×10^−3^ mm^2^/s.

Data are expressed as mean ± standard deviation.

**Fig. 2:**
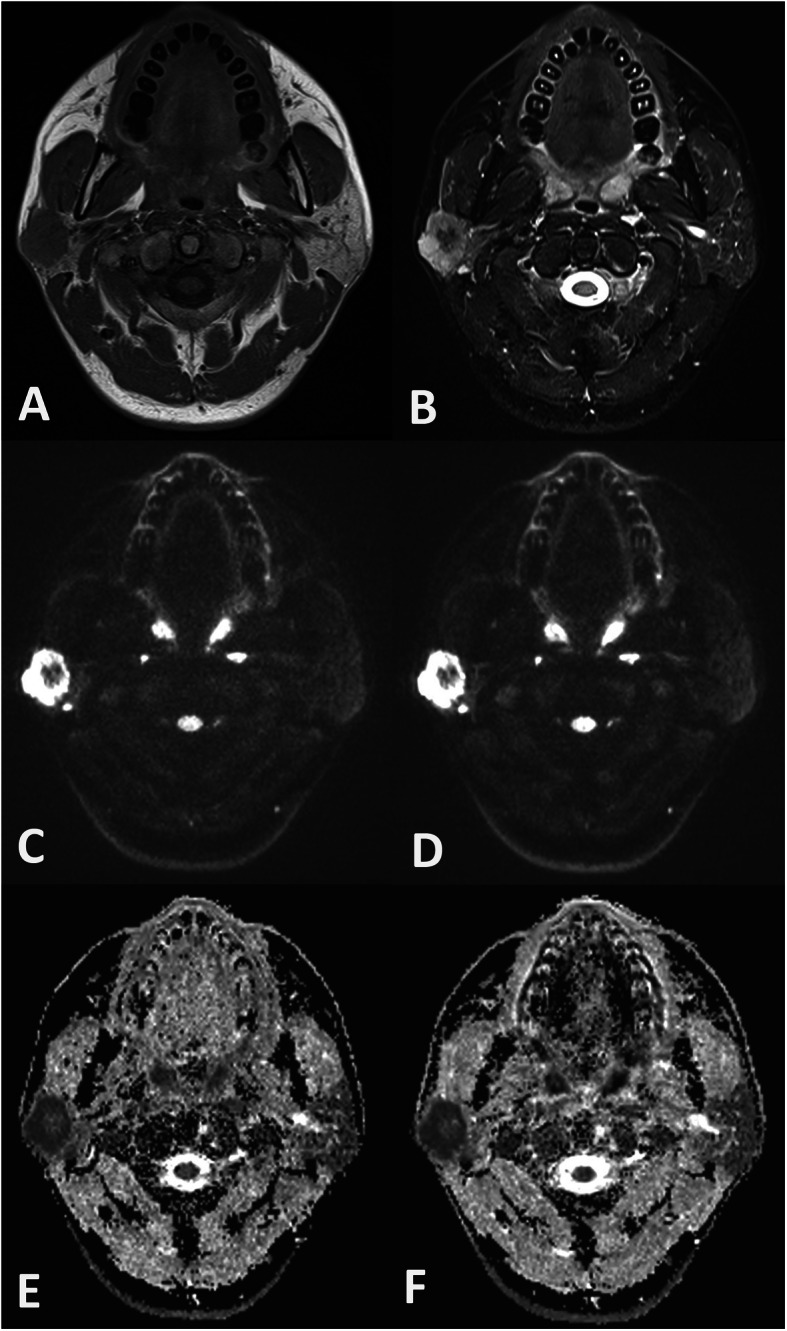
Example of SMS-RESOLVE DWI, RESOLVE DWI, and corresponding ADC maps of a 29-year-old patient with a lymphoepithelioma in the right parotid gland. The tumor showed isointensity on T1-weighted image (**a**), hyperintensity on T2-weighted image (**b**), and peripheral hyperintensity and central hypointensity on axial DWI (**c**, SMS-RESOLVE DWI; **d**, RESOLVE DWI). Image quality scores were similar for SMS-RESOLVE DWI and conventional RESOLVE DWI. The ADC value of the tumor derived from SMS-RESOLVE DWI (**e**) and RESOLVE DWI (**f**) were 0.54 × 10^− 3^ mm^2^/s and 0.52× 10^− 3^ mm^2^/s, respectively

**Fig. 3:**
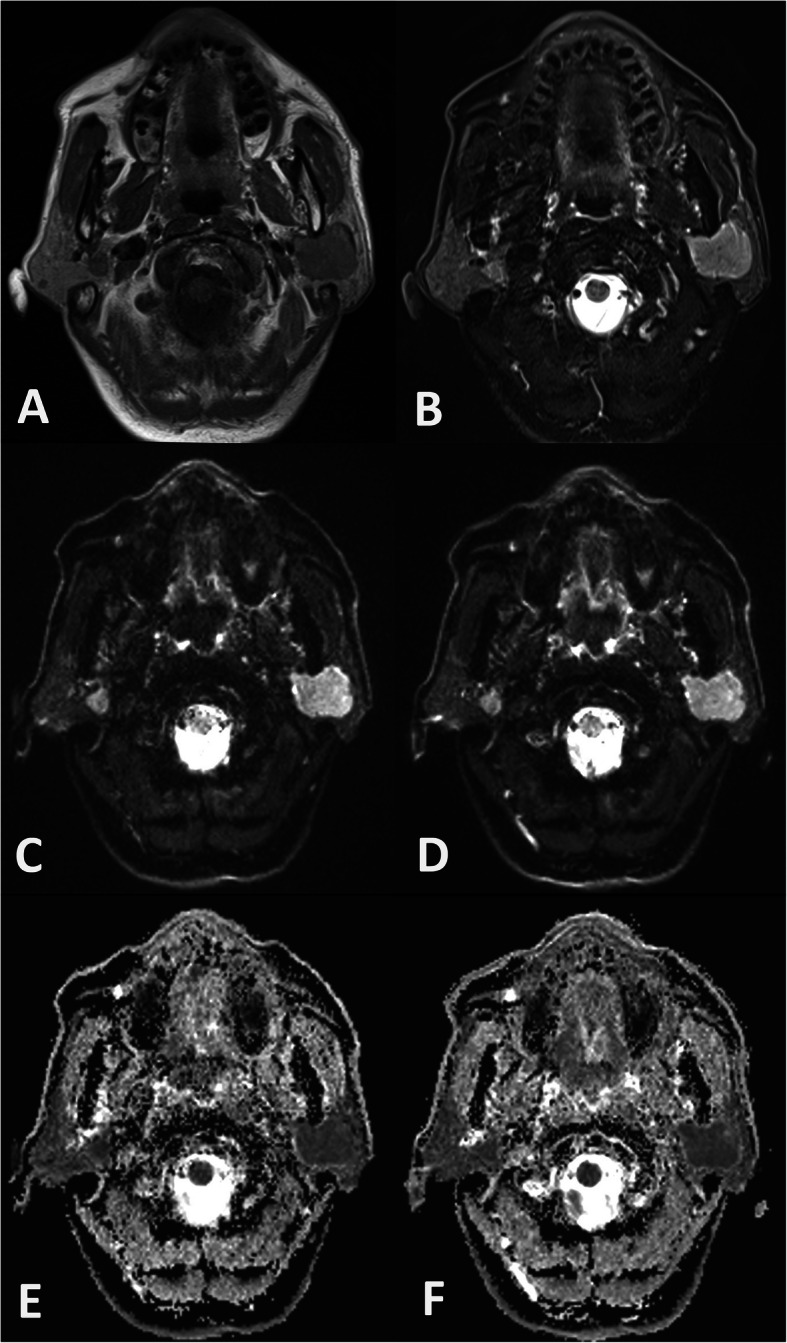
Example of SMS-RESOLVE DWI, RESOLVE DWI, and corresponding ADC maps of a 57-year-old patient with bilateral warthin tumors in parotid glands. The tumor showed slight hyperintensity on T1-weighted image (**a**), hyperintensity on T2-weighted image (**b**), and hyperintensity on axial DWI (**c**, SMS-RESOLVE DWI; **d**, RESOLVE DWI). Image quality scores were similar for SMS-RESOLVE DWI and RESOLVE DWI. The ADC value of the tumor derived from SMS-RESOLVE DWI (**e**) and RESOLVE DWI (**f**) were 0.61 × 10^− 3^ mm^2^/s and 0.62× 10^− 3^ mm^2^/s, respectively

The ICCs of the four quantitative measurements based on SMS-RESOLVE DWI ranged from 0.75–0.89, while those of the measurements based on conventional RESOLVE DWI ranged from 0.74–0.88 (Table 3). Representative images of the patients with lymphoepithelioma and warthin tumor in this study were shown in Figs. 2 and 3.

## Discussion

DWI and ADC value have been proven to be a useful imaging marker for tumor diagnosis, differentiation of histologic grade, prediction of disease survival and therapeutic monitoring in various tumors [23]. Clinical DWI is usually scanned using SS-EPI technique because of the fast scan speed, however it is prone to geometric distortion, image blurring, and image artifacts, which is more severe in head and neck region [7]. As a solution, RESOLVE technique showed remarkable advantages over SS-EPI reflected in reduced distortion and artifact [7]. However the improvement in image quality is achieved at the cost of longer acquisition time, which is a potential drawback limiting the wide application of RESOLVE DWI in clinical practice. In our study, we found that SMS-RESOLVE DWI could allow a substantial reduction of scan time while maintaining image quality with no significant difference, thereby improving the clinical applicability of SMS-RESOLVE DWI in assessing parotid gland tumors.

Two problems are usually taken into account in DWI of head and neck region. The first one is artifact, and another one is the display capability of lesions. Previous studies have demonstrated the absolute advantage of RESOLVE DWI on these two aspects, compared with SS-EPI [8, 10]. When SMS technique is combined with RESOLVE DWI, whether the reduced acquisition time would hamper such advantage is not clarified. In our study, no significant differences on the subjective scores of artifact and lesion displayed, and the objective measurements of SNR ratio and CNR ratio were observed between SMS-RESOLVE DWI and RESOLVE DWI, which was similar with the study of Filli et al. [14]. Our study result indicated that SMS technique reduced the scan time without a compromise on image quality and lesion display capability, which can increase the clinical usability of RESOLVE DWI for assessing parotid gland tumors.

One more thing we must concern is that whether the advanced DWI technique would influence the ADC value. Previously, several studies compared the ADC value derived from SS-EPI and RESOLVE DWI, and paradoxical results were obtained. Zhao et al. found that the ADC value of the sinonasal lesions on RESOVE DWI was lower than that on SS-EPI, while Bogner et al. indicated that there was no significant difference on the ADC obtained from two DWI sequences [22, 24]. In this study, we compared the ADC derived from SMS-RESOLVE DWI and RESOLVE DWI respectively, and no significant difference was found on the ADC of both masseter and tumor, which was consistent with the findings of Filli et al. [14]. Our study results indicated that SMS technique would not affect the measurement of ADC values. The derived diagnostic threshold value achieved from RESOLVE DWI based studies could be directly applied in SMS-RESOLVE DWI related study.

The scan time is 5 min and 46 s for RESOLVE DWI of parotid gland in our study, which seems too long in clinic. During our study design, we tried a highly-optimized imaging parameter. Thirty slices with a slice thickness of 3 mm were used in our study, while the slice thickness usually ranged from 4 to 6 mm in previous studies [1, 2, 25]. The voxel size was 1.1 × 1.1 × 3.0 mm^3^, and the average number of each b value was set as 2. In our opinion, DWI based on so highly-optimized parameters can provide more anatomical information on diffusion map, which is very crucial for clinical evaluation of tumor and its adjacent structures. SMS technique can reduce the scan time by nearly 2 min, increasing the applicability of so highly-optimized parameters in clinical practice.

There were several limitations should be noted. First, we did not compare our sequences with conventional SS-EPI based DWI, because the advantage of RESOLVE had been well demonstrated in previous studies [11, 12]. Second, we calculated the SNR ratio, CNR ratio and ADC values using multiple small and round ROIs those were manually placed. This method was prone to sampling bias. Third, most parotid gland tumors in our study were benign ones with clearly demarcated margin. Further large-scale studies enrolling more tumors with infiltrative margin and various histo-pathological subtypes could help us to confirm our findings, and evaluate the effect of SMS-RESOLVE DWI on diagnostic accuracy.

## Conclusion

Our study indicated that SMS technique can provide a faster RESOLVE DWI scan for parotid gland tumors without compromise in image quality in both qualitative and quantitative assessment. In this respect, SMS-RESOLVE DWI is a useful alternative to RESOLVE DWI for assessing parotid gland tumors in clinical practice.

## Data Availability

The data of this study are available from the corresponding author on reasonable request.

## Abbreviations

SMS: Simultaneous multi-slice
RESOLVE: Readout segmentation of long variable echo-trains
DWI: Diffusion-weighted imaging
ADC: Apparent diffusion coefficient
SNR: Signal-to-noise ratio
CNR: Contrast-to-noise ratio
SS-EPI: Single-shot echo-planar imaging
TR: Repetition time
TE: Echo time
ROI: Region of interest
ICC: Intra-class correlation coefficient
CI: Confidence interval
